# The Study of Plasticized Sodium Ion Conducting Polymer Blend Electrolyte Membranes Based on Chitosan/Dextran Biopolymers: Ion Transport, Structural, Morphological and Potential Stability

**DOI:** 10.3390/polym13030383

**Published:** 2021-01-26

**Authors:** Ahmad S.F.M. Asnawi, Shujahadeen B. Aziz, Iver Brevik, Mohamad A. Brza, Yuhanees M. Yusof, Saad M. Alshehri, Tansir Ahamad, M. F. Z. Kadir

**Affiliations:** 1 Chemical Engineering Section, Universiti Kuala Lumpur Malaysian Institute of Chemical & Bioengineering Technology (UniKL MICET), Alor Gajah, Malacca 78000, Malaysia; asyafiq.asnawi@s.unikl.edu.my (A.S.F.M.A.); yuhanees@unikl.edu.my (Y.M.Y.); 2 Hameedmajid Advanced Polymeric Materials Research Lab., Department of Physics, College of Science, University of Sulaimani, Qlyasan Street, Sulaimani 46001, Kurdistan Regional Government, Iraq; mohamad.brza@gmail.com; 3 Department of Civil engineering, College of Engineering, Komar University of Science and Technology, Sulaimani 46001, Kurdistan Regional Government, Iraq; 4 Department of Energy and Process Engineering, Norwegian University of Science and Technology, N-7491 Trondheim, Norway; 5 Department of Chemistry, King Saud University, P.O. Box 2455, Riyadh 11451, Saudi Arabia; alshehri@ksu.edu.sa (S.M.A.); tahamed@ksu.edu.sa (T.A.); 6 Centre for Foundation Studies in Science, University of Malaya, Kuala Lumpur 50603, Malaysia; mfzkadir@um.edu.my

**Keywords:** dextran-chitosan blend, sodium triflate, FTIR study, impedance analysis, circuit modeling, transport properties, dielectric analysis, TNM and LSV studies

## Abstract

The polymer electrolyte system of chitosan/dextran-NaTf with various glycerol concentrations is prepared in this study. The electrical impedance spectroscopy (EIS) study shows that the addition of glycerol increases the ionic conductivity of the electrolyte at room temperature. The highest conducting plasticized electrolyte shows the maximum DC ionic conductivity of 6.10 × 10^−5^ S/cm. Field emission scanning electron microscopy (FESEM) is used to investigate the effect of plasticizer on film morphology. The interaction between the electrolyte components is confirmed from the existence of the O–H, C–H, carboxamide, and amine groups. The XRD study is used to determine the degree of crystallinity. The transport parameters of number density (*n*), ionic mobility (*µ*), and diffusion coefficient (*D*) of ions are determined using the percentage of free ions, due to the asymmetric vibration (*υ_as_*(SO_3_)) and symmetric vibration (*υ_s_*(SO_3_)) bands. The dielectric property and relaxation time are proved the non-Debye behavior of the electrolyte system. This behavior model is further verified by the existence of the incomplete semicircle arc from the Argand plot. Transference numbers of ion (*t_ion_*) and electron (*t_e_*) for the highest conducting plasticized electrolyte are identified to be 0.988 and 0.012, respectively, confirming that the ions are the dominant charge carriers. The *t_ion_* value are used to further examine the contribution of ions in the values of the diffusion coefficient and mobility of ions. Linear sweep voltammetry (LSV) shows the potential window for the electrolyte is 2.55 V, indicating it to be a promising electrolyte for application in electrochemical energy storage devices.

## 1. Introduction

Natural solid polymer electrolytes (SPEs) have been widely applied to the development of energy storage devices [1]. Generally, all electrochemical devices, such as dye-sensitized solar cells, supercapacitors (SCs), batteries, and fuel cells, consist of two electrodes (a cathode and an anode) and an electrolyte. SCs or electrical double-layer capacitors (EDLCs) are certified to be one of the important power sources in certain devices, for example, memory back-ups, electrical vehicles, and digital communications, owing to their long cycle life and large power density [2]. ELDCs are prepared using two carbon electrodes separated by an electrolyte. Electrolytes have a crucial role in determining the electrochemical devices’ performance [1,3]. Numerous studies have documented the use of gel-based electrolytes in EDLCs [4,5]. Liquid electrolytes are also employed in lithium batteries [6], however liquid electrolytes have some drawbacks, such as leakage [7] and corrosion [8]. There has been plenty of research done to substitute liquid electrolytes with SPEs. Thus, numerous researchers have focused on SPE developments owing to its benefits, such as low chemical resistivity, stable potential window of more than 1 V, easy processability, good contact between electrodes, and electrolyte, very good mechanical strength, cheap, light in weight, and good ionic conductivity, which are vital for electrochemical energy storage devices [9,10,11].

In addition, the blending of polymers to produce an SPE is considered as an effective method to enhance the durability of an electrolyte [12,13,14,15]. Blending of polymers is a frequently used technique to modify the properties of the polymers which are not available in homo-polymers. Through this method, the degree of crystallinity of the polymers is reduced, due to the intermolecular interaction between blended polymers and increased convenience of site vacancy for ions hopping, which, in turn, increases the ionic conductivity of the electrolytes [12,13,14,15]. This will be beneficial for future energy storage device applications [12,13,14,15]. Natural polymers used in this field will minimally impact the environment, due to their biodegradable and other eco-friendly properties [16]. All these will aid in reducing the weaknesses of conventional batteries, including pollution [17]. In this regard, two biodegradable polymers, chitosan (CS) and dextran, were chosen to be used in this study. CS is obtained through a deacetylation process of chitin and has a few properties, such as biodegradable and biocompatible, which make it a suitable candidate to be explored as a polymer host [18,19]. The hydroxyl, acetamido, and amino groups in the structure of CS provide interaction sites within the electrolyte system [20,21]. Dextran is a natural polymer that is produced by the fermentation process of *leuconostocmesenteroides* bacteria, which have been widely explored in this area of study [22,23,24]. Moreover, various oxygen groups at the linkages of 1,6,-α-D-glucopyranosidic bond in the polymer chain of dextran help in increasing the conductivity [22]. Normally, dextran is used in the medical area, for example, as a drug carrier, blood substitute, in bone curing, and plasma modification. In the dextran structure, the presence of hydroxyl group ensures the polymer to be used as an ionic conductor [25].

Meanwhile, the selection of proton providers is also important to enhance the ionic conductivity of the system because the good performance of energy devices is highly reliant on the conductivity value of the electrolyte. Sodium-based salt is also found to give an equivalent overall performance in terms of conductivity (~10^−4^ S/cm) and stability as the salt that contains NH_4_^+^ ion [1,12] and Mg^2+^ ion [26,27], and it can also be an alternative to lithium-based salts that might affect the environment [28]. The addition of sodium triflate (NaTf) salt was reported to increase the conductivity of polyvinyl alcohol-based electrolytes from 4.87 × 10^−6^ S/cm to 2.31 × 10^−3^ S/cm at room temperature [29]. Poy et al. [30] also studied the effect of sodium-based salt on their two different electrolyte systems (sodium trifluoromethanesulfonimide (NaTFSI) and sodium trifluoromethanesulfonate (NaOTF)) and found that the conductivity of the systems was increased from 2.34 × 10^−4^ S cm^−1^ to 1.79 × 10^−3^ S cm^−1^ as the concentration of salt increased from 10 wt. % to 50 wt. %. Our previous work reported a study on dielectric properties and relaxation dynamics of the CS-NaTf electrolytes, and it was revealed that rising temperature from 303 K to 363 K could also enhance the conductivity of CS-NaTf electrolytes [31]. Furthermore, the incorporation of plasticizer can also further increase the ionic conductivity, as well as other significant parameters, such as the amorphous and thermal properties [32]. This is because the plasticizer, such as glycerol, promotes dissociation of ions, which contributes to a high performance of the electrolytes, especially for future applications [33,34]. The glycerol can dissociate more salts and disrupt hydrogen bonding between polymer chains. Thus, this improves the amorphous phase of the prepared samples, which acts as a pathway for ions conduction [24,28]. Additionally, more free ions will be available for conduction [33,34]. Glycerol also has been acknowledged and proven to be a good plasticizer for polymer electrolytes [26,35,36]. Herein, the blend of CS –dextran (60:40) wt.% is selected to be the polymer host in the present work, due to the promising characteristics discussed earlier in this section [24,37]. This work is focusing on the effect of different concentrations of glycerol on the chitosan /dextran-NaTf electrolyte system by using several characterization techniques. Table 1 lists symbols and their corresponding physical significances.

## 2. Methodology

### 2.1. Sample Preparation

All reagents used in this work were obtained from Sigma-Aldrich (Kuala Lumpur, Malaysia) and used to prepare electrolyte samples without further purification. Firstly, dextran (0.4 g) and CS (0.6 g) were separately dissolved in 1 wt.% of acetic acid (50 mL) for about 1.5 h at room temperature. The dextran and CS solutions were then mixed together homogeneously for 3 h. Sodium triflate (NaTf) salt (40 wt.%) was added to the dextran-CS blend solution and constantly stirred until the salt has completely dissolved. Glycerol, as a plasticizer in the system, was added at a specific concentration according to those tabulated in Table 2 and then correspondingly designated as those in Table 2 as well. Next, a solution casting technique was employed to obtain polymer electrolyte films. In this technique, polymer electrolytes were placed in Petri dishes for drying of samples. Then, the further drying process was carried out by keeping the Petri dishes in a desiccator with blue silica gel to produce solvent-free samples at ambient temperature. The sample preparation processes were carried out at room temperature with ~50% relative humidity. The thickness of the electrolyte samples was in the range of 0.028–0.031 cm, which was measured using a high accuracy micrometer (Mitutoyo, Coventry, UK).

### 2.2. Impedance, Morphology, and Fourier Transform Infrared Analyses

Successfully prepared electrolytes were firstly tested using an LCR meter (HIOKI 3531 Z Hi-tester, Nagano, Japan) to study their impedance properties and measure the real (*Z_r_*) and imaginary (*Z_i_*) parts of impedance. The measurement took place at room temperature with a frequency range of 50 Hz to 5 MHz. The DC potential for the experiment was 0.04 V. In this measurement, the polymer electrolyte samples with 2 cm in diameter (measure with a Vernier) were kept between two stainless-steel blocking electrodes under spring pressure. Field emission scanning electron microscopy (FESEM) was employed using a Hitachi SU8220 (Tokyo, Japan) at 500× magnification. The morphology of the samples was studied using the FESEM technique. On the other hand, the study on the interaction of the different components of the electrolytes, including polymers, salt, and plasticizer, was conducted using Fourier transform infrared (FTIR) spectroscopy. A Spotlight 400 Perkin–Elmer spectrometer was employed for this analysis with a resolution of 1 cm^−1^ (450–4000 cm^−1^). A deconvolution technique was used to extract any overlapping peaks. Correction of baseline and curve fitting were performed based on a Gaussian-Lorentzian function.

### 2.3. Transference Number Measurement (TNM) and Linear Sweep Voltammetry (LSV) Measurement

**Figure 1:**
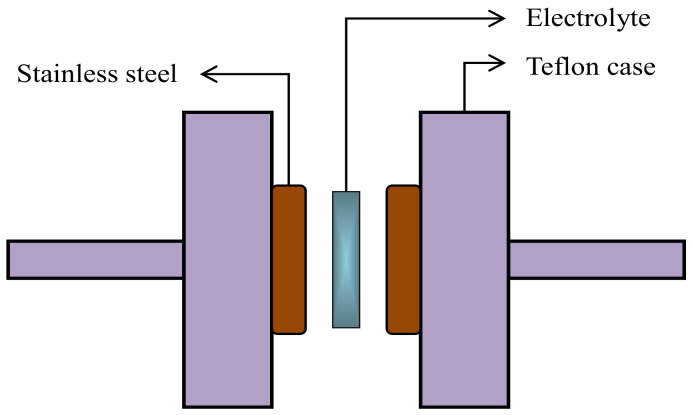
Schematic illustration of the electrolyte-electrodes arrangement for LSV analysis.

Electrochemical properties of the SPEs were initially investigated by TNM using a V&A Instrument (DP3003) (V&A Instrument, Shanghai, China) connected with a digital DC power supply. The circuit diagram for the TNM measurement was shown in our previous work, as reported in Reference [38]. This method was used to determine the ionic transference number (*t_ion_*) and the electronic transference number (*t_e_*). A working voltage of 0.20 V was applied at ambient temperature. Moreover, it is also important to identify the electrochemical stability and breakdown voltage of an electrolyte. For this purpose, LSV was conducted using a potentiostat (DY2300) (Neware, Shenzhen, China) with a scan rate of 10 mV/s. Figure 1 shows the schematic illustration of the electrodes and electrolyte arrangement for the LSV analysis, where only the highest conducting electrolyte was utilized. Stainless-steel was used as a reference, working, and counter (auxiliary) electrodes.

## 3. Result and Discussion

### 3.1. Ionic Conductivity Studies

**Figure 2:**
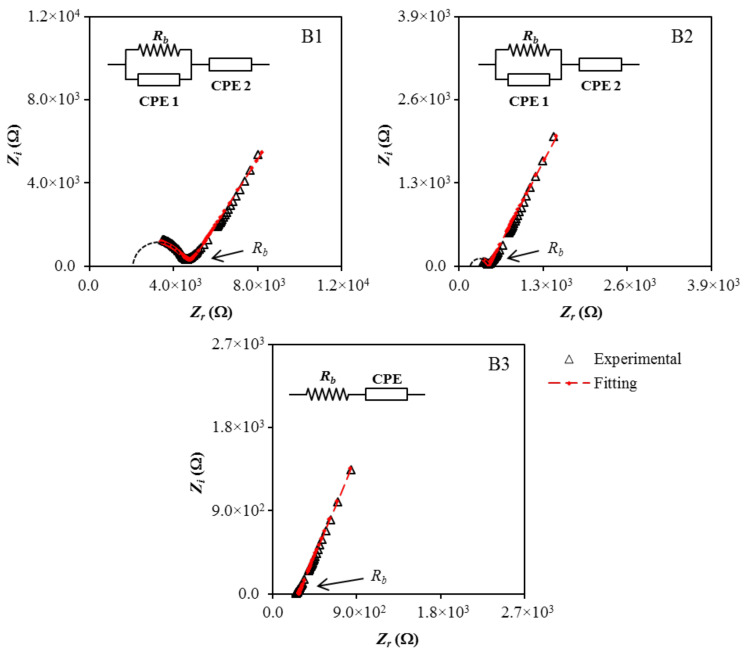
Cole–Cole plots for (**B1**), (**B2**), and (**B3**) at room temperature.

Cole–Cole plots of (B1, B2, and B3) at (stainless-steel electrodes) are shown together with the fitted curves in Figure 2. In each case, the electrical impedance spectroscopy (EIS) data were fitted to an equivalent circuit consisting of two capacitors arising from the constant phase element (CPE) of the immobile polymer chains, and a resistor is the bulk resistance (*R_b_*) of the system, as presented in the inset of Figure 2 [39]. For the B1 and B2 electrolytes, the parallel arrangement of *R_b_* and CPE1 yielded a semicircular arc observed in both Cole–Cole plots signify the charge conductivity within the electrolytes. The spike/tail at the low frequency region in the EIS plots represents the charge accumulation during the polarization process, due to the diffusion mechanism of the system [40,41].

The diameter of the semicircle in B1 is found to become smaller with the addition of glycerol and disappeared in the B3 electrolyte. The fall in the *R_b_* value with increasing plasticizer content is interconnected to the ability of glycerol to detach more NaTf salt and disrupt hydrogen bond between polymer chains. This improves the amorphous phase of the polymer electrolyte, which acts as a pathway for ion conduction, and also increases the number of movable ions. Consequently, the *R_b_* value will drop and give rise to the conductivity of the polymer electrolyte. The impedance of CPE (*Z_CPE_*) can be expressed using the following equations [42,43]:

(1) $$Z_{CPE}=\frac{1}{C\omega^{p}}\left[\cos\left(\frac{\pi p}{2}\right)-i\sin\left(\frac{\pi p}{2}\right)\right]$$

where *C* is the capacitance of CPE, *ω* is the angular frequency, and *p* is the deviation of the plot from the axis. The real and imaginary parts of the impedance, *Z_r_* and *Z_i_* of the B1 and B2 electrolytes, which consist of both semicircle and spike, can be expressed using the following equations.

(2) $$Z_{r}=\frac{R_{b}C_{1}\omega^{p_{1}}\cos\left(\frac{\pi p_{1}}{2}\right)+R_{b}}{2R_{b}C_{1}\omega^{p_{1}}\cos\left(\frac{\pi p_{1}}{2}\right)+R_{b}^{2}C_{1}^{2}\omega^{2p_{1}}+1}+\frac{\cos\left(\frac{\pi p_{2}}{2}\right)}{C_{2}\omega^{p_{2}}}$$

(3) $$Z_{i}=\frac{R_{b}^{2}C_{1}\omega^{p_{1}}\sin\left(\frac{\pi p_{1}}{2}\right)}{2R_{b}C_{1}\omega^{p_{1}}\cos\left(\frac{\pi p_{1}}{2}\right)+R_{b}^{2}C_{1}^{2}\omega^{2p_{1}}+1}+\frac{\sin\left(\frac{\pi p_{2}}{2}\right)}{C_{2}\omega^{p_{2}}}$$

where *p*_2_ and *p*_1_ are the deviation of the spike from the horizontal axis and deviation semicircle from the vertical axis, respectively. The capacitances at high and low frequency are represented as *C*_1_ and *C*_2_, respectively. For the B3 electrolyte that only has the spike and the *R_b_* is connected in series with CPE, the impedance can be expressed as:

(4) $$Z_{r}=\frac{\cos\left(\frac{\pi p}{2}\right)}{C\omega^{p}}+R_{b}$$

(5) $$Z_{i}=\frac{\sin\left(\frac{\pi p}{2}\right)}{C\omega^{p}}$$

Equations (1)–(3) were used for fitting the EIS data to an equivalent circuit for the B1 and B2 electrolytes, while Equations (1), (4), and (5) were used for fitting the EIS data to an equivalent circuit for the B3 electrolyte. The equations were also used to determine the fitting parameters (CPE1 and CPE2) and measuring the *R_b_* values precisely. The determined *R_b_* values and calculated CPE values for the B1, B2, and B3 electrolytes are tabulated in Table 3. The growth of CPE2 values with the increases of glycerol content clarified the boost of the number of ions in the electrolytes, which increase the availability for electrode polarization, hence, increasing the capacitance value at low frequency [44]. This also contributes to a better dissociation and mobility of ions, which, therefore, increases the ionic conductivity of the electrolytes [45].

Subsequently, the ionic conductivity (*σ*) values can be calculated using Equation (6) that is beneficial to show the electrical properties of the electrolytes. The calculation involves the *R_b_* values from Table 3 and also the thickness (*t*) and surface area (*A*) of the electrolytes. The *A* of the stainless-steel electrode is 2.01 cm^2^ and the *t* of the films were shown in Section 2.1.

Table 4 lists the obtained conductivity values for the electrolytes in this work.

(6) $$\sigma =\frac{t}{A\times R_{b}}$$

The conductivity of the B1 electrolyte with 12 wt.% of glycerol is found to increase from 3.22 × 10^−6^ S/cm to 6.10 × 10^−5^ S/cm when 42 wt.% glycerol was added in B3 electrolyte. According to Marf et al. [46], the ionic conductivity of the electrolytes depends on the flexibility of the polymer chain and also the mobility of ions, and the use of glycerol as a plasticizer for the system would ease these factors. The highest conductivity value obtained by the B3 electrolyte would be appropriate for the ion-conducting device applications, which necessitate an electrolyte with conductivity series from 10^−3^ to 10^−5^ S/cm [47].

**Figure 3:**
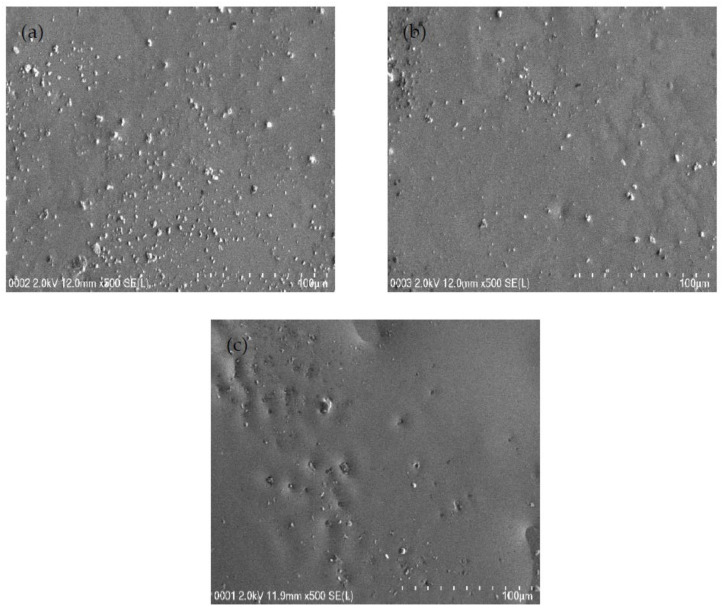
FESEM images for (**a**) B1, (**b**) B2, and (**c**) B3 electrolytes.

Field emission scanning electron micrographs were obtained at a 500× magnification for each system to support the EIS results, and the micrograph for each system is shown in Figure 3a–c. The white structures represent the protruding salts within the surface of the samples in Figure 3a–c. When there is the inclusion of 14 and 28 wt.% glycerol into the electrolyte system, few salts appeared within the films’ surface, as discovered in Figure 3a,b. The glycerol decreases the electrostatic force among cations and anions of the salt, and thus, will produce more mobile ions [48]. The existence of a plasticizer can improve the amorphous structure of the electrolyte and improve the conductivity [49]. Thus, the high concentration of glycerol can dissociate more salts into caions and anions. As it can be observed from the field emission scanning electron micrograph in Figure 3c that protruding salt structures are not evidently visible as the glycerol concentration was increased to 42 wt.% (B3 system) in comparison to the B1 and B2 electrolyte systems. The highest plasticized sample has smooth and uniform surface morphology without any phase separation. In our previous work [50], obvious phase separation in CS:PEO blend electrolyte has been detected. The lack of phase separation in Figure 3 is a confirmation for the complete blending of CS with dextran. The field emission scanning electron micrographs are in good agreement with the results of EIS. The white structures decreased in the B3 system, and the *R_b_* value diminished, as seen in the EIS plot, while conductivity increased. It is documented that the smooth morphology appearance is related to the amorphous nature development of the electrolyte systems [51]. In the previous work [50], the combined results of EIS and morphological appearance were used to understand the structure-property relationship. The smooth surface electrolytes can aid conducting ions to transfer easily, and hence, improve the DC ionic conductivity [51].

### 3.2. FTIR and XRD Studies

**Figure 4:**
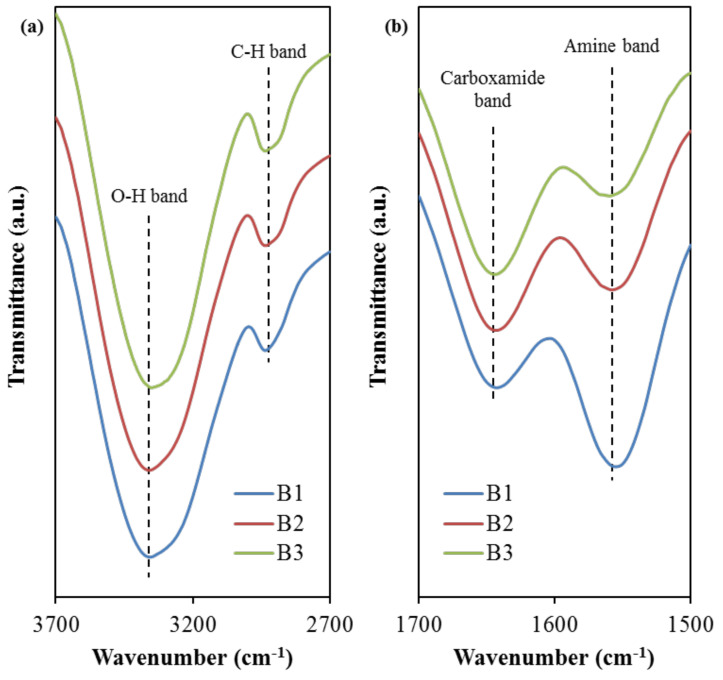
FTIR spectra at (**a**) 2700–3700 cm^−1^ and (**b**) 1500–1700 cm^−1^ band regions.

The interaction among the elements of the electrolyte, polymers, sodium salt, and glycerol can be identified from the FTIR analysis. The FTIR spectra for the selected band regions are depicted in Figure 4.

Based on Figure 4a, the peak situated at 3331 cm^−1^ is designated for the O–H band in the B1 electrolyte, which has then slightly shifted to 3332 cm^−1^ (B2) and 3333 cm^−1^ (B3). Comparable O–H band peaks were observed in the FTIR spectra of other studies using relatively similar electrolyte components, such as PVA:NaTf system at 3319 cm^−1^ [27], PVA:dextran: NH_4_I at 3332 cm^−1^ [52], and PVA: CS:ammonium bromide (NH_4_Br) at 3324 cm^−1^ [52]. The intensity variation of this band and its shifting signify a stronger interaction among blended host polymer, salt, and plasticizer in the electrolyte. This is because any changes in the FTIR spectra arise from the modifications in the vibrational and stretching modes, due to the interaction between the electrolyte components [53,54]. This increased interaction is promoting the dissociation of ions, which is beneficial for the enhancement of ionic conductivity of the electrolytes [54]. This intensity increment (see Figure 4) further supports the ionic conductivity trend, as shown in Table 4. Moreover, the C–H stretching band for the B1 electrolyte is noticed to peak at 2908 cm^−1^. As the concentration of glycerol increases, this band shifted to 2906 cm^−1^ and 2903 cm^−1^ when the B2 and B3 electrolyte was used, respectively. This range is in good agreement with 2897 cm^−1^ in Asnawi et al.’s work [55] involving poly(ethylene oxide) (PEO): Graphene oxide (GO): Ammonium triflate (NH_4_CF_3_SO_3_), and 2920 cm^−1^ in Liebeck et al.’s work [56] involving cellulose/keratin hydrolysate. According to Aziz et al. [57], the C–H stretching band at this wavenumber region is due to the presence of dextran within the electrolyte, whereby chitosan does not exhibit this band. Furthermore, Figure 4b shows the FTIR spectra in which the carboxamide band and the amine band for the B1 electrolyte are located at 1642 cm^−1^ and 1557 cm^−1^, respectively, which have then correspondingly moved to 1643 cm^−1^ and 1560 cm^−1^ when the glycerol concentration increased to 42 wt.% in B3 electrolyte. The observed positive shift shows the effect of glycerol concentration on its interaction with polymer-salt complexes. The presence of glycerol with high dielectric constant dissociates more salts to free ions, yielding more ions to interact with oxygen and nitrogen atoms in the polymer blend [54,58]. Aziz et al. [59] prepared solid polymer electrolyte chitosan:dextran:NH_4_I and used FTIR technique to study the interaction between the electrolyte elements. The authors reported 1651 and 1554 cm^−1^ for the carboxamide (O=C–NHR) and amine (NH_2_) bands, respectively, for their electrolyte systems.

**Figure 5:**
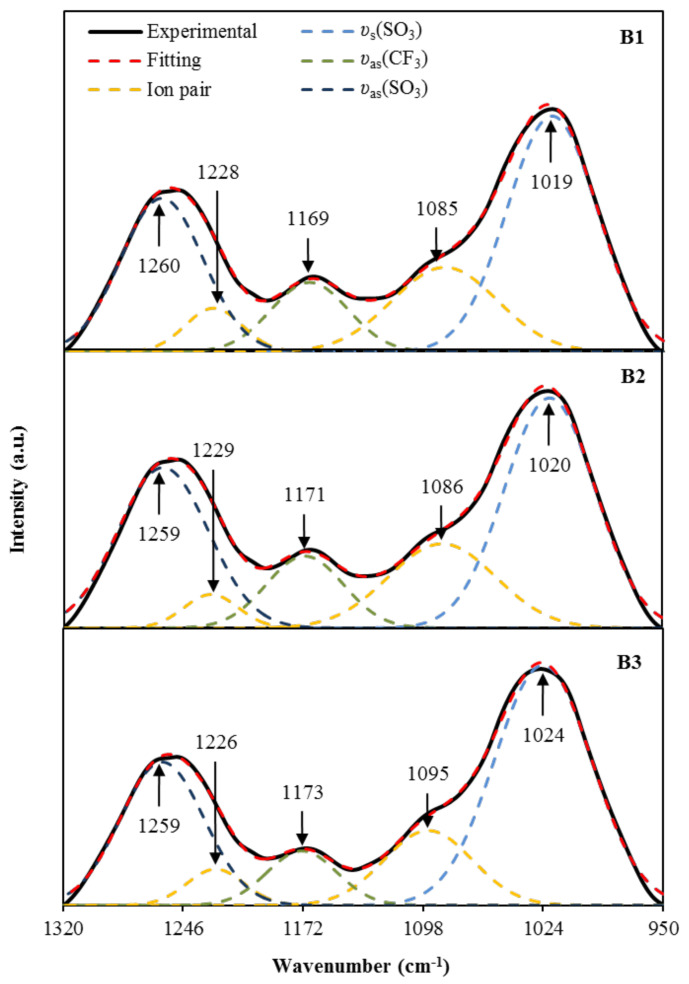
The deconvoluted FTIR spectra at 950–1320 cm^−1^ for the electrolytes.

On the other hand, the region of 950–1320 cm^−1^ is known to include a few important functional groups, such as asymmetric vibration (*υ_as_*) and symmetric vibration (*υ_s_*), associated with the trifalte anion (CF_3_SO_3_)^−^ of NaTf salt [60,61]. According to Jeya et al. [60], the two bands at 1259 cm^−1^ and 1027 cm^−1^ are attributable to free ions *υ_as_*(SO_3_) and *υ_s_*(SO_3_). The authors also mentioned that the free ions’ peak will split into two, one at a lower and another at a higher wavenumber. This region is also used to determine the transport parameters; number density (*n*), ionic mobility (*µ*), and diffusion coefficient (*D*) based on the percentage of free ions, and it is highly correlated to the conductivity of the electrolytes [62]. The deconvoluted FTIR spectra for the region of 950–1320 cm^−1^ are shown in Figure 5. For B1 electrolyte, the free ions’ peak are identified at 1260 cm^−1^ and 1019 cm^−1^, while the ion pairs peak are identified at 1228 cm^−1^ and 1085 cm^−1^. For B2 electrolyte, the free ions’ peak are observed at 1259 cm^−1^ and 1020 cm^−1^; whereas, the ion pairs peak are identified at 1229 cm^−1^ and 1086 cm^−1^. For B3 electrolyte, the free ions’ peak are determined at 1259 cm^−1^ and 1024 cm^−1^, while the ion pairs peak are observed at 1226 cm^−1^ and 1095 cm^−1^. As the intensity of these free ions peaks is increased, the formation of ion pairs will occur. Besides, the *υ_as_*(CF_3_) band obtained by the electrolytes in this work, which is at 1169–1173 cm^−1^ is similar to 1165–1173 cm^−1^ reported by Ranjana et al. for the Polyvinylidenefluoride-co-hexafluoropropylene: Polymethylmethacrylate:NaTf electrolyte system [61]. The percentage of free ions can be determined using the following relation.

(7) $$\mathrm{Free}\;\mathrm{ions}\;\%=\frac{\mathrm{Area}\;\mathrm{of}\;\mathrm{free}\;\mathrm{ions}’\;\mathrm{peak}}{\mathrm{Total}\;\mathrm{area}\;\left(\mathrm{free}\;\mathrm{ions}\;\mathrm{peak}+\mathrm{ion}\;\mathrm{pairs}\;\mathrm{peak}\right)}\times 100$$

The peak intensity of free ions, due to the *υ_as_*(SO_3_) and *υ_s_*(SO_3_), is observed to increase as the concentration of glycerol increases. The corresponding free ion percentage was 75% for B1 electrolyte, 77% for B2 electrolyte, and 80% for B3 electrolyte. The increase of free ions percentage as the concentration of glycerol is increased, showing a similar trend with the ionic conductivity results achieved by the electrolytes. From the deconvolution of FTIR spectra in Figure 5, the calculated transport parameters using the following equations are tabulated in Table 5.

(8) $$n=\frac{M\times N_{A}}{V_{Total}}\times \left(\mathrm{free}\;\mathrm{ion}\;\%\right)$$

(9) $$\mu =\frac{\sigma }{ne}$$

(10) $$D=\frac{\mu kT}{e}$$

where *N_A_* is Avogadro’s number, *M* represents the number of moles of glycerol, *σ* is DC conductivity, which is shown in Table 4, *T* is the temperature (298 K), *K* is the Boltzmann constant (1.38 × 10^−23^ J/K), and *e* is the elementary charge. *V_Total_* is the total volume of the polymer electrolytes.

The calculated transport parameters in Table 5 show that the addition of glycerol into the electrolyte from B1 to B3 has increased both *n* and *μ* by one order of magnitude, and almost a four-fold increase in *D*. These results are harmonized with the ionic conductivity pattern because the high number density directly contributes to a high conductivity value [63]. Pritam et al. [64] also reported that the dependency of ionic conductivity on the transport parameters in the system of PEO/PVP-NaNO_3_ that investigated using the FTIR approach.

**Figure 6:**
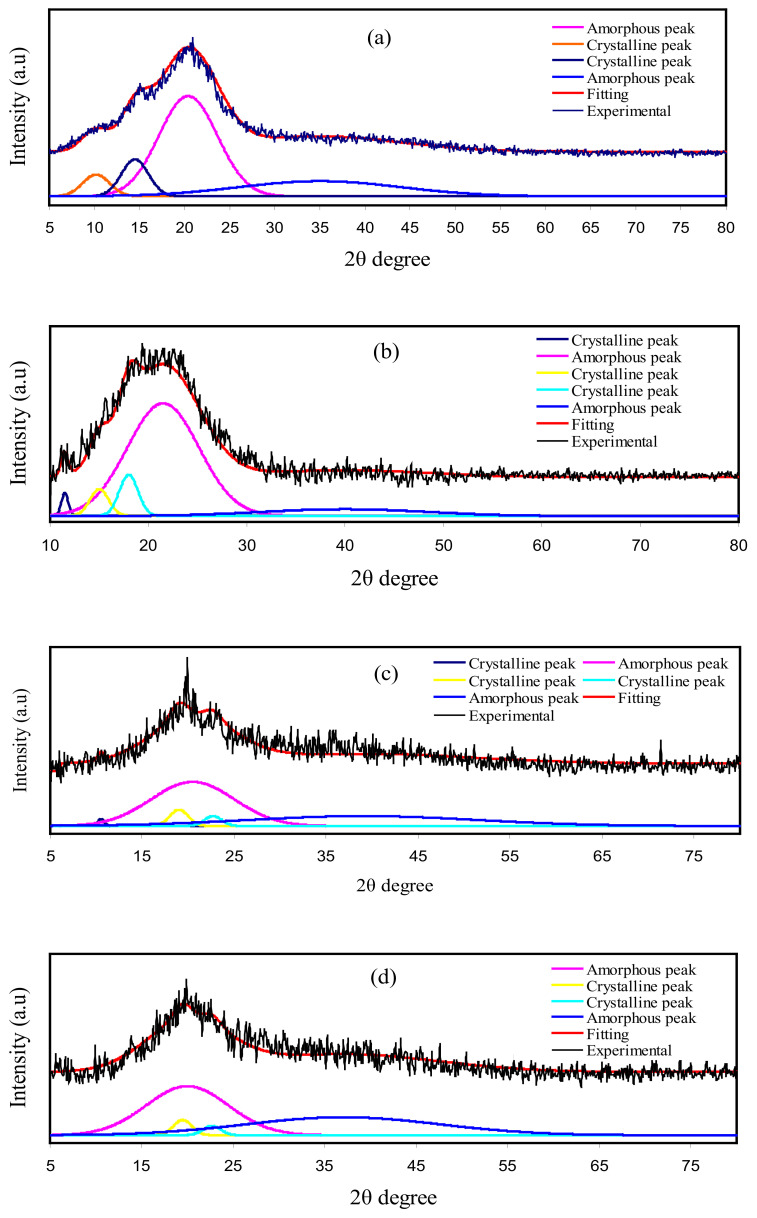
XRD for (**a**) pure CS, (**b**) CS Dex blend, (**c**) B1, and (**d**) B3 electrolytes.

XRD for pure CS, CS: dextran, and CS: dextran: NaTf: glycerol at room temperature are shown in Figure 6. The CS has crystalline peaks at the 2θ values of 10.1°, 15.1°, and 20.9° [65,66] (Figure 6a), while dextran possesses two hallows at 2θ values of 18° and 23°, as indicated in the previous work [67]. In the current work, two hallows and smaller crystalline peaks were observed in the XRD pattern of CS: dextran (Figure 6b). It is interesting that (as shown by the broad hallows) CS: dextran is not as crystalline as pure CS and its structure is almost amorphous [68,69]. Based on earlier studies, the amorphous nature of polymer electrolyte is related to broad diffraction peaks [70,71]. To determine the crystallinity degree, it is vital to deconvolute the XRD pattern for each film to find the areas of the crystalline and amorphous peaks [71]. The degree of crystallinity (Xc) was determined using Equation (11), and shown in Table 6 [72]:

(11) $$X_{c}=\left[\frac{A_{c}}{\left(A_{T}\right)}\right]\times 100\%$$

where *A_T_* and *A_C_* are the total area of crystalline and amorphous peaks and crystalline peaks’ area, respectively.

Meanwhile, it has been observed in this study, when glycerol was included, the CS: dextran peaks showed intensity decline, and its wide nature was improved, as shown in Figure 6c,d. Such observations confirm that the polymer electrolyte has an amorphous structure, which improves better conductivity by enhancing ionic diffusivity. Moreover, the NaTf salt experiences full dissociation in the polymer electrolyte that there is not any peak associated with pure NaTf. The hydrogen bonding elimination among the polymer chains is a probable reason for the reduction and broadening in intensity, indicating the amorphous phase prevalence within the samples [73].

### 3.3. Dielectric and Electric Modulus Analysis

**Figure 7:**
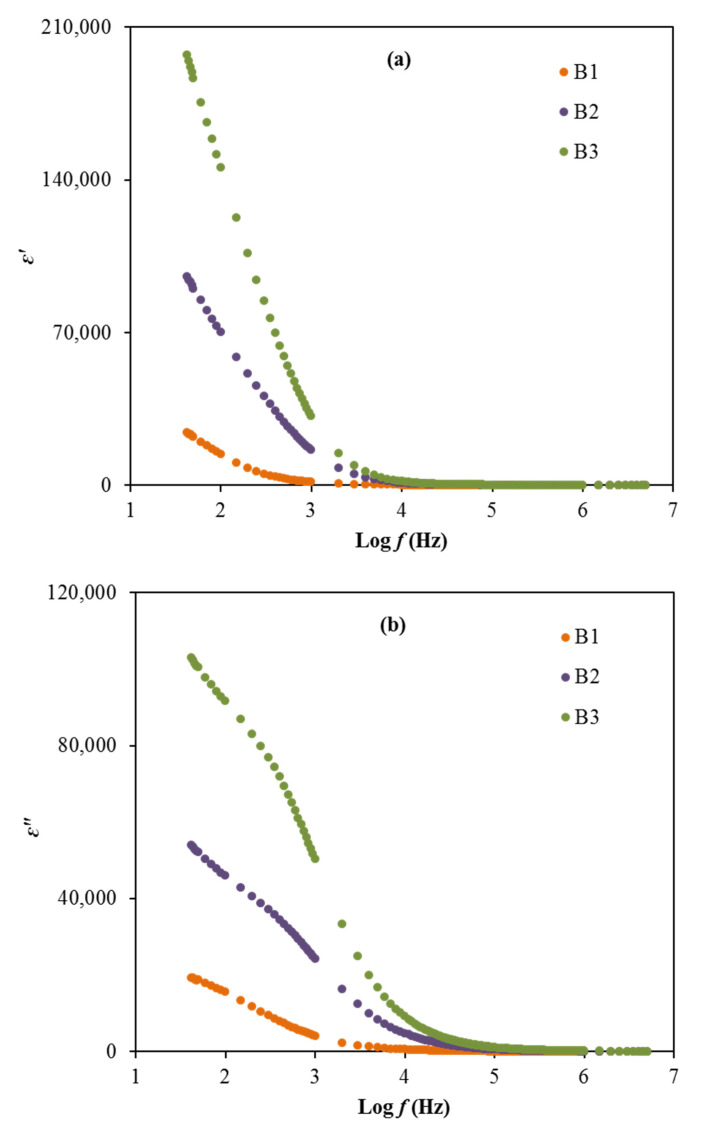
The plot of (**a**) ε’ and (**b**) ε” versus frequency for the electrolytes.

The polarization effects, as well as the conductivity behavior, can be further studied based on dielectric properties, including the dielectric loss (ε″) and dielectric constant (ε′), which represent the amount of energy loss and charge stored, respectively, during the movement of ions [74]. The *Z_r_* and *Z_i_* data were extracted from the EIS data and then used to determine the ε′ and ε″. These dielectric parameters can be calculated using Equations (12) and (13) [26,31].

(12) $$\varepsilon \prime =\frac{Z_{i}}{\left(Z_{r}^{2}+Z_{i}^{2}\right)C_{o}\omega }$$

(13) $$\varepsilon \prime \prime =\frac{Z_{r}}{\left(Z_{r}^{2}+Z_{i}^{2}\right)C_{o}\omega }$$

where *C_o_* stands for the vacuum capacitance and *ω* is the angular frequency. The plot of ε′ and ε″ for the electrolytes at room temperature can be observed in Figure 7a,b.

Based on Figure 7, the order of dielectric constant and dielectric loss decreases from B3 to B2 to B1 in the low frequency region. The high dielectric values obtained in this work, especially for the B3 electrolyte, explains that the accumulation of charge carriers can cause the polarization of electrodes and also the space charge effect within the system [55,75]. The lack of any peak in Figure 7 due to dielectric relaxation designates the system is predominantly due to polymer relaxation segments in their ionic conductivity [76]. However, the values of ε′ and ε″ for the electrolytes are observed to reduce as the frequency increases and remain at a constant value, which caused by the periodic reversal of the electric field that occurred rapidly between the electrodes [77]. Generally, the dielectric plots show the effects of glycerol concentration in the electrolytes on the ε′ and ε″ values where the employment of glycerol as a plasticizer help to enhance these properties, which are also harmonized with the conductivity and transport parameters trends. The non-Debye model is suitable to represent the conductivity behavior of the electrolytes.

Furthermore, the frequency dependence of dielectric loss tangent (tan *δ*) can be determined for the electrolytes to further understand the relaxation processes of the electrolytes. As mentioned above, the *Z_r_* and *Z_i_* data have been obtained from the EIS data and then used to find the ε’ and ε” data. The dielectric parameters are used to determine the tan *δ*. The tan *δ* is a ratio of energy disperse to energy stored in a periodical field, which is also known as the dissipation factor [78]. The tan *δ* is determined using the relation below [78].

(14) $$\tan\;\delta =\;\frac{\varepsilon^{\prime \prime }}{\varepsilon \text{′}}$$

**Figure 8:**
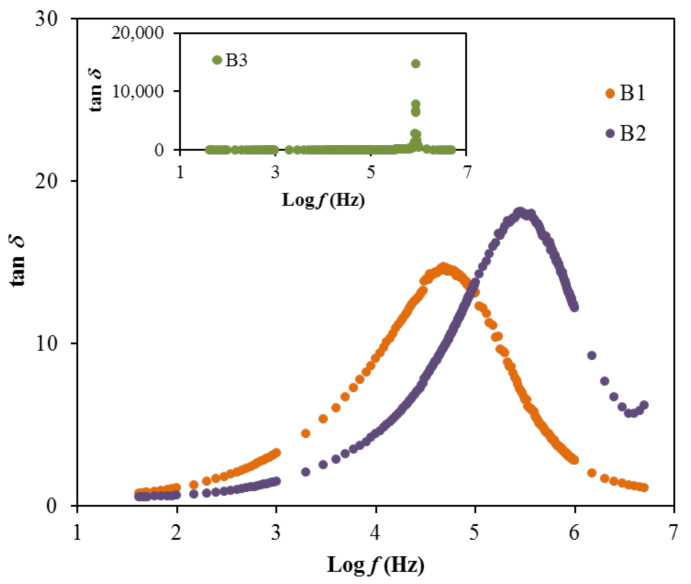
The tan *δ* plot for the electrolytes.

The relaxation processes of polymer materials are precisely studied via loss tangent peaks. The dipoles in the polymer electrolytes can be illustrated based on the dielectric relaxation [79,80]. Figure 8 shows the dielectric relaxation of the loss tan δ vs. frequency plot for each film at room temperature. In the figure, there is a shift to a region of the high frequency of the loss tangent peak, showing the occurrence of dielectric relaxation. One finding of this work is that induced or permanent dipoles cause the conductivity and dielectric relaxation peaks. It was also indicated that induced diploes hide the polarization relaxation of mobile charge carriers in the materials [79,80]. The peaks observed in Figure 8 describe the translational ion dynamics that are related to the mobile ions’ conductivity relaxation. This is an advantage for the ions transport in the segmental motion of the electrolytes [80]. The tan *δ* is noticed to increase when the frequency increases, due to the dominant amount of active element (ohmic) compared to the reactive element (capacitive). Following this event, the reduction of tan *δ* at a higher frequency is probably due to the independency of the active element to the frequency, which causes the reactive element to increase accordingly [81]. The relaxation process of the electrolytes signified by the tan *δ* plot proposes the non-Debye behavior of the system [82]. Furthermore, the tan *δ* value that is located at the highest frequency can be designated as the tan *δ* maximum, tan *δ_max_* value, which is valuable to control the angular frequency, *ω_peak_* of the relaxation peak. Therefore, the relaxation time (*t_r_*) of each electrolyte can be calculated by inversing the *ω_peak_* (1/*ω_peak_*). The calculated *t_r_* values are listed in Table 7.

**Figure 9:**
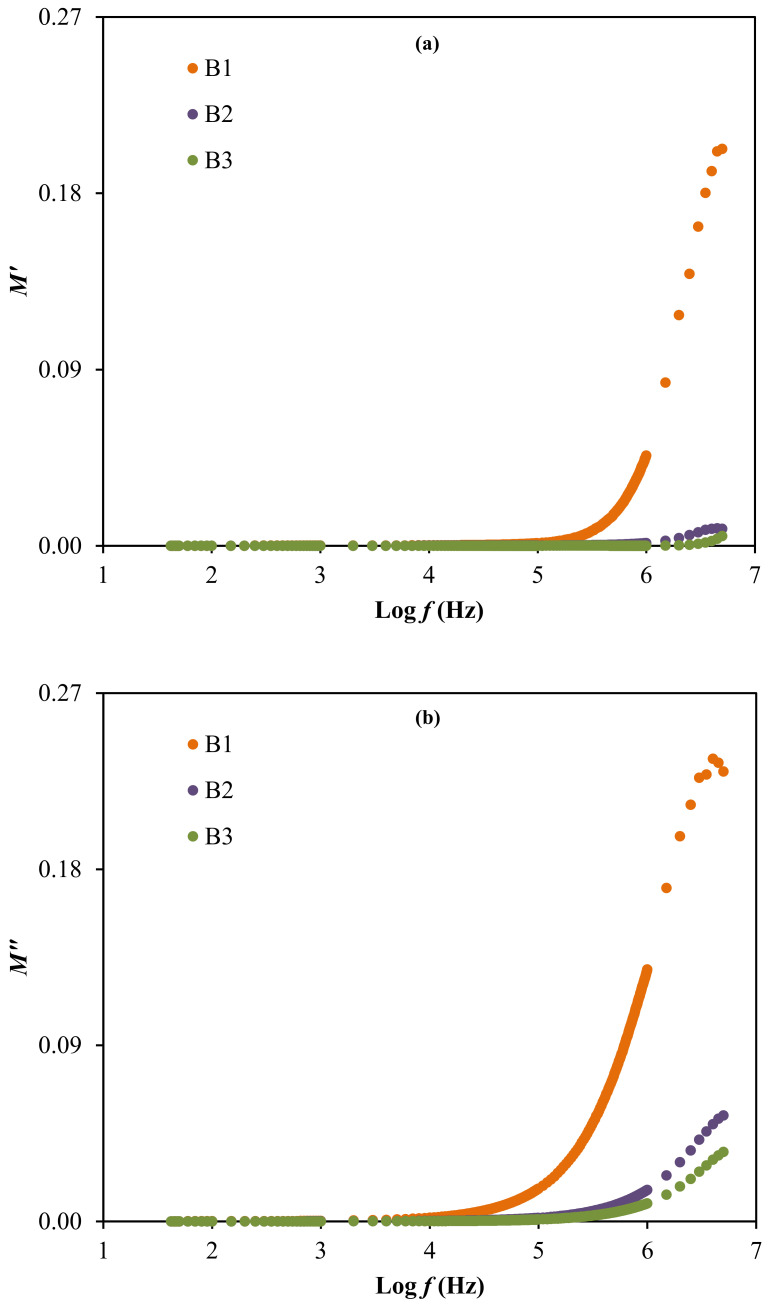
The electrical modulus of (**a**) *M*′ and (**b**) *M*″ for the electrolytes.

The result of relaxation time is noticed to reduce as the ionic conductivity of the electrolytes increases, as observed in Table 4 and Table 7. This is because the ions are mostly attached to the polymer chain during their movement in the segmental motion that is beneficial for the hopping process between the conduction sites [83]. The lowest relaxation time for the electrolyte with the highest concentration of glycerol verifies the faster ion dynamics within the system [84]. Vahini et al. [85] also reported the low relaxation time, which was responsible for the high ionic conductivity value. In addition, the electrical modulus study can be used to further describe the polarization suppression effect of the system. The real (*M*′) and imaginary (*M*″) parts of electrical modulus at room temperature were evaluated using Equation (15) and Equation (16) [86], respectively, and the results are plotted in Figure 9 [86].

(15) $$M\prime =\omega C_{o}Z_{i}$$

(16) $$M\prime \prime =\omega C_{o}Z_{r}$$

**Figure 10:**
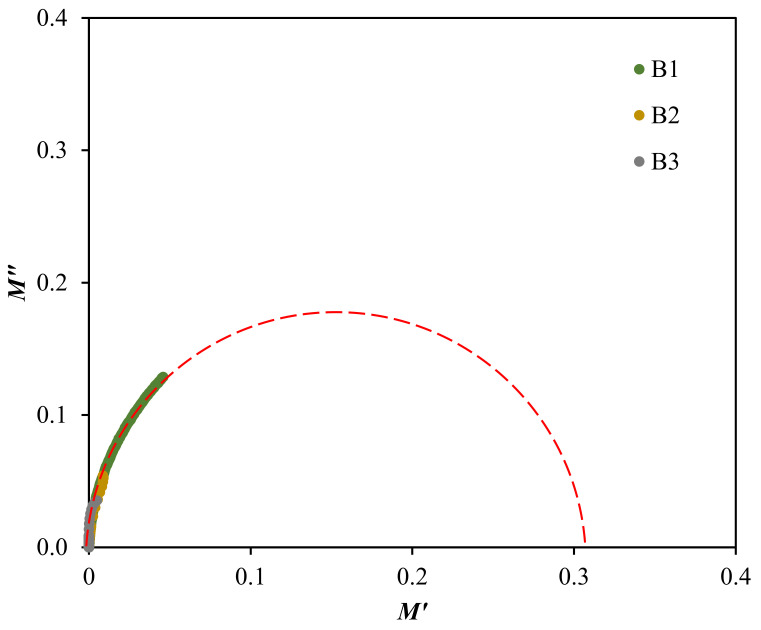
Argand plot at room temperature for the electrolytes.

The electrical modulus analysis [87] can also be used to investigate the dielectric properties caused by the relaxation of ions because the electrode polarization is interrelated to the development of charges near the electrodes. Based on Figure 9, the electrical modulus values are observed to stay near zero at low frequency in both plots. The long tail detected at low frequency proposes the capacitive behavior of the electrolytes where the strong electrode polarization occurs without any dispersion [36,78]. Along with the frequency, the *M*′ and *M*″ values of the electrolytes are observed to increase, due to the bulk effect. The highest conducting electrolyte obtained the lowest electrical modulus values at high frequencies. A similar observation is reported by Mustafa et al. [87] and Saminatha Kumaran et al. [88]. The presence of peaks in both modulus plots explained the enhancement of polymer chain flexibility, signifying that the electrolytes are considered as good ionic conductors [89,90]. Furthermore, the plot of *M*″ versus *M*′ or also known as Argand plot for the electrolytes at room temperature, is depicted in Figure 10.

The Argand plot in Figure 10 illustrates a single incomplete semicircle, which is extrapolated to suit each electrolytes curve. This deformed semicircle is generally an indicator for the broad relaxation processes within the system and also reveals the non-Debye behavior [91]. The ionic conductivity of the electrolytes is found to be directly influenced with the radius of arc in the Argand plot, where the smaller arc contributes to a higher conductivity value [92]. This relationship is also related to the resistivity of the electrolytes. This is correlated to Equation (16), since the curve of the highest conducting electrolyte, B3 is closer towards the origin [71].

### 3.4. Electrochemical Investigation

**Figure 11:**
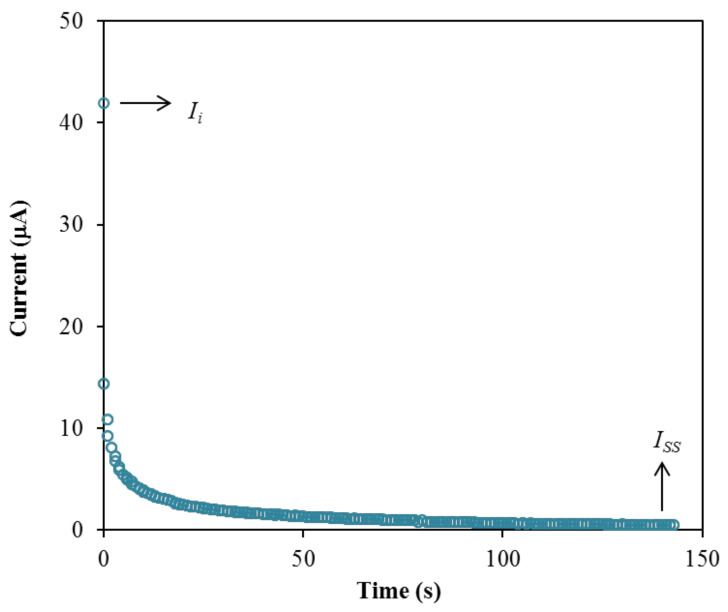
Polarization current versus time for the B3 electrolyte.

The performance of the highest conducting electrolyte, B3 is further tested using transference number measurement (TNM) and linear sweep voltammetry (LSV) to identify its suitability for the application in energy devices. Firstly, TNM analysis was conducted at room temperature to investigate the contribution of particular charge species within the electrolyte, either ions or electron, which can be determined through the polarization of current against time as plotted in Figure 11.

The plot in Figure 11 exhibits a drastic drop of initial current (*I_i_*) and then reaches a steady state of current (*I_ss_*) at 100th s and onwards. During the initial current drop, the drift of ions is equivalent to the diffusion of ions where the ions are blocked by the characteristics of the stainless-steel electrode [93]. Only electrons are allowed to transport during this phase. The large value of current in the beginning is because the ions and electrons are involved. In the beginning, the cations and anions move in opposite directions toward the surface of the electrodes from the bulk of the electrolyte to form the double-layer at the electrode-electrolyte interfaces, and polarization occurs. The polarization of the cell occurs when it reaches the steady state, while transport of the rest of the currents is only due to electrons [94]. This is why the electron transference number (*t_e_*) can be determined using stainless-steel electrodes. Subsequently, the steady state of current is developed by the formation of diffusion layers on the electrode interface, where a large resistance of a passive ionic layer was produced [79,95]. Therefore, ions were not involved in the current flow in this region [79,95]. When the ions are totally reduced, a steady state of current flow that is due to electrons is obtained [96]. The transference numbers of the ion (*t_ion_*) and electron (*t_e_*) for the B3 electrolyte are calculated using the following equations.

(17) $$t_{ion}=\frac{I_{i}-I_{ss}}{I_{i}}$$

(18) $$t_{e}=\frac{I_{ss}}{I_{i}}$$

**Figure 12:**
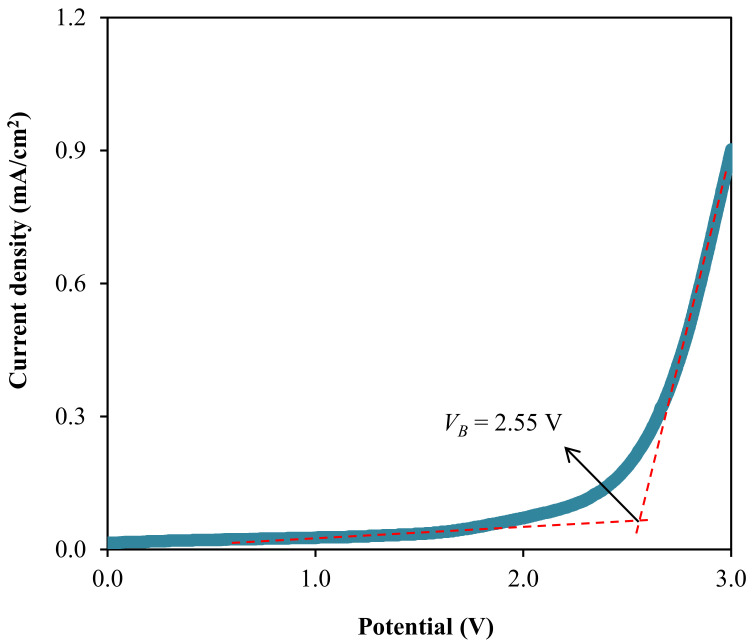
Linear sweep curve for the B3 electrolyte.

The B3 electrolyte achieved a high *t_ion_* value of 0.988, and signifies that ions were dominant in the system [97]. The *t_e_* value obtained is 0.012. According to Shukur et al. [98], the contributor of the carrying charge of a system is due to the ions if the *t_ion_* value is near to unity. A similar observation is reported by Mohan et al. [99] and Tang et al. [100]. The high *t_ion_* value can be used to further examine the contribution of ions in the B3 electrolyte, which can be calculated using the following equations [101]:

(19) $$t_{ion}=\frac{D_{+}}{D_{+}+D_{-}}=\;\frac{D_{+}}{D}$$

(20) $$D=\frac{\mu k_{b}T}{e}=D_{+}+D_{-}$$

(21) $$t_{ion}=\frac{\mu_{+}}{\mu_{+}+\mu_{-}}=\;\frac{\mu_{+}}{\mu }$$

(22) $$\mu =\frac{\sigma }{ne}=\mu_{+}+\mu_{-}$$

where *D_+_* and *D_−_* represent the diffusion coefficient of cation and anion; while *µ_+_* and *µ_−_* are the ionic mobility of cation and anion, respectively. The calculation is based on the transport parameters listed in Table 5. From Equation (19), $D_{+}=t_{ion}\times D$, $D_{+}$ can be calculated and by using Equation (20), $D_{-}=D-D_{+}$, *D*− can be obtained. The calculated values of *D_+_* and *D_−_* for the B3 electrolyte are found to be 9.50 × 10^−11^ and 1.15 × 10^−12^ cm^2^ s^−1^, respectively. Similarly, using Equations (21) and (22), $\mu_{+}$ and $\mu_{-}$ were founded to be 3.70 × 10^−9^ and 4.49 × 10^−11^ cm^2^ V^−1^ s^−1^, respectively. From these results, it can be concluded that the cationic values are more significant than those of anionic for both *D* and *µ*. Therefore, in polymer electrolytes, researchers have mainly focused on cations rather than anions. The ionic conductivity is found to be reliant on these two parameters (*D* and *µ*) [6]. Hafiza et al. [102] also reported a similar observation for the influence of ions on *D* and *µ* values. The diffusion coefficient values achieved in this work is analogous to the earlier studies where the diffusion coefficient is in the range from 10^−13^ to 10^−8^ [96,103,104,105]. Furthermore, another vital characteristic of an electrolyte is the maximum working voltage. This measurement is significant to identify the breakdown voltage (*V_B_*) of an electrolyte that can withstand that can be acquired from the LSV analysis [106]. The LSV curve for the highest conducting electrolyte, B3 (at room temperature), is presented in Figure 12 at a scan rate of 10 mV/s.

Based on Figure 12, the LSV curve of the B3 electrolyte does not experience the current density growth before reaching 2.55 V. This illustrates worthy electrochemical stability within the system electrolyte system up to that potential [107]. Therefore, the breakdown voltage of 2.55 V is possessed by the B3 electrolyte. Moreover, Liew et al. were shown that the addition of plasticizer to the PVA-CH_3_COONH_4_ electrolyte-enhanced the *V_B_* value of the system from 1.80 V to 2.20 V and observed to has a promising performance in the energy devices [108]. The *V_B_* value determined in this work is high enough to be applied in the electrochemical devices that are normally run at the operating voltage of 1.0 V [109].

## 4. Conclusions

The polymer electrolytes of chitosan/dextran-NaTf with three different glycerol concentrations were been prepared. From the impedance study, the addition of glycerol has increased the ionic conductivity of the electrolyte at room temperature. The B3 electrolyte achieved the highest conductivity of 6.10 × 10^−5^ S/cm. It was revealed by the FESEM method that at higher glycerol concentration, that the films have a smooth and homogenous surface morphology. The interaction of the components within the electrolytes was confirmed from the presence of the O-H, C-H, carboxamide, and amine groups. The transport parameters were identified using the percentage of free ions, due to the *υ_as_*(SO_3_) and *υ_s_*(SO_3_) bands. The determined X_c_ revealed that the amorphous phase improved with plasticizer addition. The dielectric properties and relaxation time verified the non-Debye behavior of the electrolyte system. This behavior model was further proved by the presence of an incomplete semicircle arc from the Argand plot. The ions ere investigated to be the most dominant element in the B3 electrolyte with a *t_ion_* value of 0.988 and *t_e_* of 0.012. The *t_ion_* value was used to further examine the contribution of ions in the ionic mobility and diffusion coefficient values. From the determined values of *D_+_*, *D_−_*, $\mu_{+}$ and $\mu_{-}$, it can be concluded that the cationic values were more significant than those of anionic for both *D* and *µ*. The breakdown voltage of the B3 electrolyte was observed at 2.55 V from the LSV analysis, which may be a promising electrolyte for the electrochemical energy devices applications.

## Figures and Tables

**Table 1:** Symbols and their corresponding physical significances.

Symbols	Physical Significances
SPE	Solid polymer electrolyte
EDLC	Electric double-layer capacitor
CPE	Constant phase element in F
*R_b_*	Bulk resistance in Ω
*t_ion_*	Ion transference number
*t_e_*	Electron transference number
*σ*	DC ionic conductivity in S/cm
*t*	Thickness of the sample in cm
*A*	Area of the sample in cm^2^
*N_A_*	Avogadro’s number
*M*	Number of moles of glycerol
*T*	Temperature in K
*K_B_*	Boltzmann constant in J/K
*V_Total_*	Total volume of the polymer electrolytes
*D*	Diffusion coefficient of ions in cm^2^ s^−1^
*µ*	Mobility of ions in cm^2^ V^−1^ s^−1^
*n*	Number density of ions in cm^−3^
*P*	Deviation from the axes
*C*	Capacitance in F
*ω*	Angular frequency in Hz
*C_o_*	Vacuum capacitance in F
ε′	Dielectric constant
ε″	Dielectric loss
*I_i_*	Initial current
*I_ss_*	Steady state current
*µ* ^+^	Mobility of cations in cm^2^ V^−1^ s^−1^
*µ* ^−^	Mobility of anions in cm^2^ V^−1^ s^−1^
*D* ^+^	Diffusion coefficient of cations in cm^2^ s^−1^
*D* ^−^	Diffusion coefficient of anions in cm^2^ s^−1^

**Table 2:** Designation of the polymer electrolytes with different amounts of glycerol.

Glycerol (wt.%)	Designation
12	B1
28	B2
42	B3

**Table 3:** Room temperature circuit element for the electrolytes.

Electrolyte	*R_b_* (Ω)	CPE1 (F)	CPE2 (F)
B1	(4.79 ± 1.2) × 10^3^	(4.00 ± 1.3) × 10^−9^	(2.86 ± 0.7) × 10^−6^
B2	(3.21 ± 1.0) × 10^2^	(2.86 ± 1.1) × 10^−9^	(3.70 ± 1.0) × 10^−6^
B3	(2.53 ± 0.8) × 10^2^	-	(5.41 ± 1.2) × 10^−6^

**Table 4:** Room temperature ionic conductivity for the electrolytes.

Electrolyte	*σ* (S/cm)
B1	3.22 × 10^−6^
B2	4.80 × 10^−5^
B3	6.10 × 10^−5^

**Table 5:** Transport parameters of the electrolytes at room temperature.

Electrolyte	Number Density, *n* (cm^−3^)	Ionic Mobility, *μ* (cm^2^ V^−1^ s^−1^)	Diffusion Coefficient, *D* (cm^2^ s^−1^)
B1	2.13 × 10^22^	9.44 × 10^−10^	2.42 × 10^−11^
B2	8.96 × 10^22^	3.35 × 10^−9^	8.59 × 10^−11^
B3	1.02 × 10^23^	3.75 × 10^−9^	9.61 × 10^−11^

**Table 6:** The degree of crystallinity obtained from deconvoluted XRD pattern.

Electrolyte	Degree of Crystallinity (%)
Pure CS	15.97
CS Dex	13.85
B1	7.96
B3	5.52

**Table 7:** The relaxation time, *t_r_* for the electrolytes.

Electrolyte	*t_r_* (s)
B1	3.32 × 10^−6^
B2	5.49 × 10^−7^
B3	1.81 × 10^−7^

## Data Availability

The data presented in this study are available on request from the corresponding author.

## References

[1] Yusof Y.M., Shukur M.F., Illias H.A., Kadir M.F.Z. (2014). Conductivity and electrical properties of corn starch-chitosan blend biopolymer electrolyte incorporated with ammonium iodide. Phys. Scr..

[2] Kim W.J., Kim D.W. (2008). Sulfonated poly(ether ether ketone) membranes for electric double layer capacitors. Electrochim. Acta.

[3] Hu X., Muchakayala R., Song S., Wang J., Chen J., Tan M. (2018). Synthesis and optimization of new polymeric ionic liquid poly(diallydimethylammonium) bis(trifluoromethane sulfonyl)imde based gel electrolyte films. Int. J. Hydrogen Energy.

[4] Zhang B., Liang J., Xu C.L., Wei B.Q., Ruan D.B., Wu D.H. (2001). Electric double-layer capacitors using carbon nanotube electrodes and organic electrolyte. Mater. Lett..

[5] Chang J., Park M., Ham D., Ogale S.B., Mane R.S., Han S.H. (2008). Liquid-Phase synthesized mesoporous electrochemical supercapacitors of nickel hydroxide. Electrochim. Acta.

[6] McBreen J., Lee H.S., Yang X.Q., Sun X. (2000). New approaches to the design of polymer and liquid electrolytes for lithium batteries. J. Power Sources.

[7] Mahendran O., Chen S.Y., Chen-Yang Y.W., Lee J.Y., Rajendran S. (2005). Investigations on PMMA-PVdF polymer blend electrolyte with esters of dibenzoic acids as plasticizers. Ionics.

[8] Staiti P., Minutoli M., Lufrano F. (2002). All solid electric double layer capacitors based on Nafion ionomer. Electrochim. Acta.

[9] Singh R., Singh P.K., Singh V., Bhattacharya B. (2019). Quantitative analysis of ion transport mechanism in biopolymer electrolyte. Opt. Laser Technol..

[10] Kumar S., Prajapati G.K., Saroj A.L., Gupta P.N. (2019). Structural, electrical and dielectric studies of nano-composite polymer blend electrolyte films based on (70–x) PVA–x PVP–NaI–SiO_2_. Phys. B Condens. Matter.

[11] Singh R., Bhattacharya B., Gupta M., Khan Z.H., Tomar S.K., Singh V., Singh P.K. (2017). Electrical and structural properties of ionic liquid doped polymer gel electrolyte for dual energy storage devices. Int. J. Hydrogen Energy.

[12] Moniha V., Alagar M., Selvasekarapandian S., Sundaresan B., Boopathi G. (2018). Conductive bio-polymer electrolyte iota-carrageenan with ammonium nitrate for application in electrochemical devices. J. Non Cryst. Solids.

[13] Riess I. (2000). Polymeric mixed ionic electronic conductors. Solid State Ion..

[14] Parameswaran V., Nallamuthu N., Devendran P., Nagarajan E.R., Manikandan A. (2017). Electrical conductivity studies on Ammonium bromide incorporated with Zwitterionic polymer blend electrolyte for battery application. Phys. B Condens. Matter.

[15] Bakar N.Y.A., Muhamaruesa N.H.M., Aniskari N.A.B., Isa M.I.N.M. (2015). Electrical studies of carboxy methycellulose-chitosan blend biopolymer doped dodecyltrimethyl ammonium bromide solid electrolytes. Am. J. Appl. Sci..

[16] Samsudin A., Isa M. (2012). Structural and electrical properties of carboxy methylcellulose-dodecyltrimethyl ammonium bromide-based biopolymer electrolytes system. Int. J. Polym. Mater. Polym. Biomater..

[17] Samsudin A.S., Isa M.I.N. (2012). Ion conducting mechanism of carboxy methylcellulose doped with ionic dopant salicylic acid based solid polymer electrolytes. Int. J. Appl. Sci. Technol..

[18] Aziz S.B., Hamsan M.H., Kadir M.F.Z., Woo H.J. (2020). Design of polymer blends based on chitosan: POZ with improved dielectric constant for application in polymer electrolytes and flexible electronics. Adv. Polym. Technol..

[19] Stepniak I., Galinski M., Nowacki K., Wysokowski M., Jakubowska P., Bazhenov V.V., Leisegang T., Ehrlich H., Jesionowski T. (2016). A novel chitosan/sponge chitin origin material as a membrane for supercapacitors-preparation and characterization. RSC Adv..

[20] Zulkifli A.M., Said N.I.A.M., Aziz S.B., Hisham S., Shah S., Bakar A.A., Abidin Z.H.Z., Tajuddin H.A., Sulaiman L., Brza M.A. (2020). Electrochemical characteristics of phthaloyl chitosan based gel polymer electrolyte for dye sensitized solar cell application. Int. J. Electrochem. Sci..

[21] Khiar A.S.A., Puteh R., Arof A.K. (2006). Characterizations of chitosan-ammonium triflate (NH_4_CF_3_SO_3_) complexes by FTIR and impedance spectroscopy. Phys. Status Solidi Appl. Mater. Sci..

[22] Sarwat F., Ahmed N., Aman A., Qader S.A.U. (2013). Optimization of growth conditions for the isolation of dextran producing *Leuconostoc* spp. from indigenous food sources. Pak. J. Pharm. Sci..

[23] Vettori M.H.P.B., Franchetti S.M.M., Contiero J. (2012). Structural characterization of a new dextran with a low degree of branching produced by *Leuconostoc mesenteroides* FT045B dextransucrase. Carbohydr. Polym..

[24] Aziz S.B., Hamsan M.H., Kadir M.F.Z., Karim W.O., Abdullah R.M. (2019). Development of polymer blend electrolyte membranes based on chitosan: Dextran with high ion transport properties for EDLC application. Int. J. Mol. Sci..

[25] Telegeev G., Kutsevol N., Chumachenko V., Naumenko A., Telegeeva P., Filipchenko S., Harahuts Y. (2017). Dextran-Polyacrylamide as Matrices for Creation of Anticancer Nanocomposite. Int. J. Polym. Sci..

[26] Hamsan H.M., Aziz S., Kadir M.F.Z., Brza M.A., Karim W. (2020). The study of EDLC device fabricated from plasticized magnesium ion conducting chitosan based polymer electrolyte. Polym. Test..

[27] Anilkumar K.M., Jinisha B., Manoj M., Jayalekshmi S. (2017). Poly(ethylene oxide) (PEO)—Poly(vinyl pyrrolidone) (PVP) blend polymer based solid electrolyte membranes for developing solid state magnesium ion cells. Eur. Polym. J..

[28] Gong S.D., Huang Y., Cao H.J., Lin Y.H., Li Y., Tang S.H., Wang M.S., Li X. (2016). A green and environment-friendly gel polymer electrolyte with higher performances based on the natural matrix of lignin. J. Power Sources.

[29] Farah N., Ng H.M., Numan A., Liew C.W., Latip N.A.A., Ramesh K., Ramesh S. (2019). Solid polymer electrolytes based on poly(vinyl alcohol) incorporated with sodium salt and ionic liquid for electrical double layer capacitor. Mater. Sci. Eng. B Solid State Mater. Adv. Technol..

[30] Poy S.Y., Bashir S., Omar F.S., Saidi N.M., Farhana N.K., Sundararajan V., Ramesh K., Ramesh S. (2020). Poly (1-vinylpyrrolidone-co-vinyl acetate) (PVP-co-VAc) based gel polymer electrolytes for electric double layer capacitors (EDLC). J. Polym. Res..

[31] Aziz S.B., Mamand S.M. (2018). The Study of dielectric properties and conductivity relaxation of ion conducting chitosan: NaTf based solid electrolyte. Int. J. Electrochem. Sci..

[32] Liang S., Huang Q., Liu L., Yam K.I. (2009). Microstructure and molecular interaction in glycerol plasticized chitosan/poly(vinyl alcohol) blending films. Macromol. Chem. Phys..

[33] Yusof Y.M., Kadir M.F.Z. (2016). Electrochemical characterizations and the effect of glycerol in biopolymer electrolytes based on methylcellulose-potato starch blend. Mol. Cryst. Liq. Cryst..

[34] Kadir M., Salleh N., Hamsan M., Aspanut Z., Majid N., Shukur M. (2018). Biopolymeric electrolyte based on glycerolized methyl cellulose with NH_4_Br as proton source and potential application in EDLC. Ionics (Kiel).

[35] Shukur M.F., Hamsan M.H., Kadir M.F.Z. (2019). Investigation of plasticized ionic conductor based on chitosan and ammonium bromide for EDLC application. Mater. Today Proc..

[36] Asnawi A.S.F.M., Aziz S.B., Nofal M., Abdulwahid R.T., Kadir M.F.Z., Hamsan M.H., Brza M.A., Yusof Y.M., Abdilwahid R.T. (2020). Glycerolized Li^+^ Ion conducting chitosan-based polymer electrolyte for energy storage EDLC device applications with relatively high energy density. Polymers.

[37] Kadir M., Hamsan M. (2018). Green electrolytes based on dextran-chitosan blend and the effect of NH_4_SCN as proton provider on the electrical response studies. Ionics (Kiel).

[38] Brza M.A., Aziz S.B., Anuar H., Ali F., Hamsan M.H., Kadir M.F.Z. (2020). Metal framework as a novel approach for the fabrication of electric double layer capacitor device with high energy density using plasticized poly(vinyl alcohol): Ammonium thiocyanate based polymer electrolyte. Arab. J. Chem..

[39] Kumar M., Tiwari T., Chauhan J.K., Srivastava N. (2014). Erratum: UnderstanDing the ion dynamics and relaxation behavior from impedance spectroscopy of NaI doped Zwitterionic polymer system. Mater. Res. Express.

[40] Gohel K., Kanchan D.K. (2018). Ionic conductivity and relaxation studies in PVDF-HFP:PMMA-based gel polymer blend electrolyte with LiClO_4_ salt. J. Adv. Dielectr..

[41] Misenan M., Khiar A. (2018). Conductivity, dielectric and modulus studies of methylcellulose-NH_4_TF polymer. Eurasian J. Biol. Chem. Sci. J..

[42] Teo L.P., Buraidah M.H., Nor A.F.M., Majid S.R. (2012). Conductivity and dielectric studies of Li_2_SnO_3_. Ionics (Kiel).

[43] Aziz S.B., Marif R.B., Brza M.A., Hamsan M.H., Kadir M.F.Z. (2019). Employing of Trukhan model to estimate ion transport parameters in PVA based solid polymer electrolyte. Polymers.

[44] Awasthi P., Das S. (2019). Reduced electrode polarization at electrode and analyte interface in impedance spectroscopy using carbon paste and paper. Rev. Sci. Instrum..

[45] Lee D.K., Allcock H.R. (2010). The effects of cations and anions on the ionic conductivity of poly[bis(2-(2-methoxyethoxy)ethoxy)phosphazene] doped with lithium and magnesium salts of trifluoromethanesulfonate and bis(trifluoromethanesulfonyl)imidate. Solid State Ion..

[46] Marf A.S., Aziz S.B., Abdullah R.M. (2020). Plasticized H^+^ ion-conducting PVA:CS-based polymer blend electrolytes for energy storage EDLC application. J. Mater. Sci. Mater. Electron..

[47] Aziz S.B., Brza M.A., Brevik I., Hafiz M.H., Asnawi A.S.F.M., Yusof Y.M., Abdulwahid R.T., Kadir M.F.Z. (2020). Blending and characteristics of electrochemical double-layer capacitor device assembled from plasticized proton ion conducting chitosan: Dextran: NH_4_PF_6_ polymer electrolytes. Polymers (Basel).

[48] Brza M.A., Aziz B.S., Anuar H., Dannoun E.M.A., Ali F., Abdulwahid R.T., Kadir M.F.Z. (2020). The study of EDLC device with high electrochemical performance fabricated from proton ion conducting PVA-Based polymer composite electrolytes plasticized with glycerol. Polymers.

[49] Hamsan M.H., Shukur M.F., Kadir M.F.Z. (2017). NH_4_NO_3_ as charge carrier contributor in glycerolized potato starch-methyl cellulose blend-based polymer electrolyte and the application in electrochemical double-layer capacitor. Ionics.

[50] Aziz S.B., Abdullah R.M. (2018). Crystalline and amorphous phase identification from the tanδ relaxation peaks and impedance plots in polymer blend electrolytes based on [CS:AgNt]x:PEO(x-1) (10 ≤ x ≤ 50). Electrochim. Acta.

[51] Mobarak N.N., Ahmad A., Abdullah M.P., Ramli N., Rahman M.Y.A. (2013). Conductivity enhancement via chemical modification of chitosan based green polymer electrolyte. Electrochim. Acta.

[52] Aziz S.B., Brza M.A., Hamsan M.H., Kadir M.F.Z., Muzakir S.K., Abdulwahid R.T. (2020). Effect of ohmic-drop on electrochemical performance of EDLC fabricated from PVA: Dextran: NH_4_I based polymer blend electrolytes. J. Mater. Res. Technol..

[53] Yusof Y.M., Illias H.A., Kadir M.F.Z. (2014). Incorporation of NH_4_Br in PVA-chitosan blend-based polymer electrolyte and its effect on the conductivity and other electrical properties. Ionics (Kiel).

[54] Salleh N.S., Aziz S.B., Aspanut Z., Kadir M.F.Z. (2016). Electrical impedance and conduction mechanism analysis of biopolymer electrolytes based on methyl cellulose doped with ammonium iodide. Ionics (Kiel).

[55] Asnawi A.S.F.M., Azli A., Hamsan M., Kadir M., Yusof Y. (2020). Electrical and infrared spectroscopic analysis of solid polymer electrolyte based on polyethylene oxide and graphene oxide blend. Malays. J. Anal. Sci..

[56] Liebeck B.M., Hidalgo N., Roth G., Popescu C., Böker A. (2017). Synthesis and characterization of methyl cellulose/keratin hydrolysate composite membranes. Polymers (Basel).

[57] Aziz S.B., Brza M.A., Hamsan H.M., Kadir M.F.Z., Abdulwahid R.T. (2020). Electrochemical characteristics of solid state double-layer capacitor constructed from proton conducting chitosan-based polymer blend electrolytes. Polym. Bull..

[58] Shukur M.F., Yusof Y.M., Zawawi S.M.M., Illias H.A., Kadir M.F.Z. (2013). Conductivity and transport studies of plasticized chitosan-based proton conducting biopolymer electrolytes. Phys. Scr..

[59] Aziz S.B., Hamsan M.H., Nofal M.M., Karim W.O., Brevik I., Brza M., Abdulwahid R.T., Al-Zangana S., Kadir M.F. (2020). Structural, impedance and electrochemical characteristics of electrical double layer capacitor devices based on chitosan: Dextran biopolymer blend electrolytes. Polymers (Basel).

[60] Jeya S., Arulsankar A., Abarna S., Sundaresan B. (2019). Effect of ionic liquids on the electrical, structural and morphological properties of P(VdF-HFP)-NaTF electrolytes. Ionics (Kiel).

[61] Ranjana P.A.B., Jeya S., Abarna S., Premalatha M., Arulsankar A., Sundaresan B. (2019). Enhancement of Na^+^ ion conduction in polymer blend electrolyte P(VdF-HFP)—PMMA-NaTf by the inclusion of EC. J. Polym. Res..

[62] Mejenom A.A., Hafiza M.N., Isa M.I.N. (2018). X-ray diffraction and infrared spectroscopic analysis of solid biopolymer electrolytes based on dual blend carboxymethyl cellulose-chitosan doped with ammonium bromide. ASM Sci. J..

[63] Aniskari N.A.B., Mohd Isa M.I.N. (2017). The effect of ionic charge carriers in 2-hydroxyethyl cellulose solid biopolymer electrolytes doped glycolic acid via FTIR-deconvolution technique. J. Sustain. Sci. Manag..

[64] Arya A., Sharma A.L. (2019). Dielectric relaxations and transport properties parameter analysis of novel blended solid polymer electrolyte for sodium-ion rechargeable batteries. J. Mater. Sci..

[65] Aziz S.B., Abidin Z.H.Z., Kadir M.F.Z. (2015). Innovative method to avoid the reduction of silver ions to silver nanoparticles in silver ion conducting based polymer electrolytes. Phys. Scr..

[66] Aziz S.B., Kadir M.F.Z., Abidin Z.H.Z. (2016). Structural, morphological and electrochemical impedance study of CS: LiTf based solid polymer electrolyte: Reformulated Arrhenius equation for ion transport study. Int. J. Electrochem. Sci..

[67] Hamsan M.H., Shukur M.F., Aziz S.B., Kadir M.F.Z. (2019). Dextran from *Leuconostoc mesenteroides*-doped ammonium salt-based green polymer electrolyte. Bull. Mater. Sci..

[68] Aziz S.B., Abidin Z.H.Z., Arof A.K. (2010). Effect of silver nanoparticles on the DC conductivity in chitosan-silver triflate polymer electrolyte. Phys. B.

[69] Yusuf S.N.F., Azzahari A.D., Yahya R., Majid S.R., Careem M.A., Arof A.K. (2016). From crab shell to solar cell: A gel polymer electrolyte based on N-phthaloylchitosan and its application in dye-sensitized solar cells. RSC Adv..

[70] Malathi J., Kumaravadivel M., Brahmanandhan G.M., Hema M., Baskaran R., Selvasekarapandian S. (2010). Structural, thermal and electrical properties of PVA-LiCF_3_SO_3_ polymer electrolyte. J. Non Cryst. Solids.

[71] Aziz S.B. (2016). Role of dielectric constant on ion transport: Reformulated Arrhenius equation. Adv. Mater. Sci. Eng..

[72] Wan Y., Creber K.A.M., Peppley B., Bui V.T. (2003). Synthesis, characterization and ionic conductive properties of phosphorylated chitosan membranes. Macromol. Chem. Phys..

[73] Smitha B., Sridhar S., Khan A.A. (2005). Chitosan-Sodium alginate polyion complexes as fuel cell membranes. Eur. Polym. J..

[74] Mohamed A.S., Shukur M.F., Kadir M.F.Z., Yusof Y.M. (2020). Ion conduction in chitosan-starch blend based polymer electrolyte with ammonium thiocyanate as charge provider. J. Polym. Res..

[75] Rani M.S.A., Ahmad A., Mohamed N.S. (2018). A comprehensive investigation on electrical characterization and ionic transport properties of cellulose derivative from kenaf fibre-based biopolymer electrolytes. Polym. Bull..

[76] Aziz S.B., Abdullah R.M., Kadir M.F.Z., Ahmed H.M. (2019). Non suitability of silver ion conducting polymer electrolytes based on chitosan mediated by barium titanate (BaTiO_3_) for electrochemical device applications. Electrochim. Acta.

[77] Ramly K., Isa M.I.N., Khiar A.S.A. (2011). Conductivity and dielectric behaviour studies of starch/PEO+ x wt-%NH_4_NO_3_ polymer electrolyte. Mater. Res. Innov..

[78] Pawlicka A., Tavares F.C., Dörr D.S., Cholant C.M., Ely F., Santos M.J.L., Avellaneda C.O. (2019). Dielectric behavior and FTIR studies of xanthan gum-based solid polymer electrolytes. Electrochim. Acta.

[79] Marf A.S., Abdullah R.M., Aziz S.B. (2020). Structural, morphological, electrical and electrochemical properties of PVA: CS-Based proton-conducting polymer blend electrolytes. Membranes (Basel).

[80] Aziz S.B., Abdullah R.M., Rasheed M.A., Ahmed H.M. (2017). Role of ion dissociation on DC conductivity and silver nanoparticle formation in PVA: AgNt based polymer electrolytes: Deep insights to ion transport mechanism. Polymers.

[81] Woo H.J., Majid S.R., Arof A.K. (2012). Dielectric properties and morphology of polymer electrolyte based on poly(ε-caprolactone) and ammonium thiocyanate. Mater. Chem. Phys..

[82] Idris N.H., Senin H.B., Arof A.K. (2007). Dielectric spectra of LiTFSI-doped chitosan/PEO blends. Ionics (Kiel).

[83] Sengwa R.J., Dhatarwal P. (2020). Predominantly chain segmental relaxation dependent ionic conductivity of multiphase semicrystalline PVDF/PEO/LiClO_4_ solid polymer electrolytes. Electrochim. Acta.

[84] Ahmed H.T., Jalal V.J., Tahir D.A., Mohamad A.H., Abdullah O.G. (2019). Effect of PEG as a plasticizer on the electrical and optical properties of polymer blend electrolyte MC-CH-LiBF_4_ based films. Results Phys..

[85] Vahini M., Muthuvinayagam M., Isa M.I.N. (2019). Preparation and characterization of biopolymer electrolytes based on pectin and NaNO3 for battery applications. Polym. Sci. Ser. A.

[86] Aziz S.B., Abdullah O.G., Hussein S.A., Ahmed H.M. (2017). Effect of PVA blending on structural and ion transport properties of CS:AgNt-based polymer electrolyte membrane. Polymers (Basel).

[87] Mustafa M.S., Ghareeb H.O., Aziz S.B., Brza M.A., Al-Zangana S., Hadi J.M., Kadir M.F.Z. (2020). Electrochemical characteristics of glycerolized PEO-based polymer electrolytes. Membranes (Basel).

[88] Saminatha Kumaran V., Ng H.M., Ramesh S., Ramesh K., Vengadaesvaran B., Numan A. (2018). The conductivity and dielectric studies of solid polymer electrolytes based on poly (acrylamide-co-acrylic acid) doped with sodium iodide. Ionics (Kiel).

[89] Fuzlin A.F., Rasali N.M.J., Samsudin A.S. (2018). Effect on ammonium bromide in dielectric behavior based alginate solid biopolymer electrolytes. IOP Conf. Ser. Mater. Sci. Eng..

[90] Singh P., Bharati D.C., Kumar H., Saroj A.L. (2019). Ion transport mechanism and dielectric relaxation behavior of PVA-imidazolium ionic liquid-based polymer electrolytes. Phys. Scr..

[91] Gohel K., Kanchan D.K. (2019). Effect of PC:DEC plasticizers on structural and electrical properties of PVDF–HFP:PMMA based gel polymer electrolyte system. J. Mater. Sci. Mater. Electron..

[92] Sundaramahalingam K., Muthuvinayagam M., Nallamuthu N. (2019). AC impedance analysis of lithium ion based PEO:PVP solid polymer blend electrolytes. Polym. Sci. Ser. A.

[93] Rani M.S.A., Ahmad A., Mohamed N.S. (2018). Influence of nano-sized fumed silica on physicochemical and electrochemical properties of cellulose derivatives-ionic liquid biopolymer electrolytes. Ionics (Kiel).

[94] Brza M.A., Aziz S.B., Anuar H., Ali F. (2020). Structural, ion transport parameter and electrochemical properties of plasticized polymer composite electrolyte based on PVA: A novel approach to fabricate high performance EDLC devices. Polym. Test..

[95] Shukur M.F., Ithnin R., Kadir M.F.Z. (2014). Protonic transport analysis of starch-chitosan blend based electrolytes and application in electrochemical device. Mol. Cryst. Liq. Cryst..

[96] Chai M.N., Isa M.I.N. (2016). Novel proton conducting solid bio-polymer electrolytes based on carboxymethyl cellulose doped with oleic acid and plasticized with glycerol. Sci. Rep..

[97] Basha S.S., Rao M.C. (2018). Spectroscopic and electrochemical properties of (1-x) [PVA/PVP]: x [MgCl_2_{6H_2_O}] blend polymer electrolyte films. Int. J. Polym. Sci..

[98] Shukur M.F., Ithnin R., Kadir M.F.Z. (2016). Ionic conductivity and dielectric properties of potato starch-magnesium acetate biopolymer electrolytes: The effect of glycerol and 1-butyl-3-methylimidazolium chloride. Ionics (Kiel).

[99] Rama Mohan K., Achari V.B.S., Rao V.V.R.N., Sharma A.K. (2011). Electrical and optical properties of (PEMA/PVC) polymer blend electrolyte doped with NaClO_4_. Polym. Test..

[100] Tang J., Muchakayala R., Song S., Wang M., Kumar K.N. (2016). Effect of EMIMBF_4_ ionic liquid addition on the structure and ionic conductivity of LiBF_4_-complexed PVdF-HFP polymer electrolyte films. Polym. Test..

[101] Monisha S., Mathavan T., Selvasekarapandian S., Benial M.F.A., Aristatil G., Mani N., Premalatha M., Vinoth Pandi D. (2017). Investigation of bio polymer electrolyte based on cellulose acetate-ammonium nitrate for potential use in electrochemical devices. Carbohydr. Polym..

[102] Hafiza M.N., Isa M.I.N. (2020). Correlation between structural, ion transport and ionic conductivity of plasticized 2-hydroxyethyl cellulose based solid biopolymer electrolyte. J. Membr. Sci..

[103] Shamsuri N.A., Zaine S.N.A., Yusof Y.M., Yahya W.Z.N., Shukur M.F. (2020). Effect of ammonium thiocyanate on ionic conductivity and thermal properties of polyvinyl alcohol-methylcellulose-based polymer electrolytes. Ionics (Kiel).

[104] Zulkifli A., Saadiah M.A., Mazuki N.F., Samsudin A.S. (2020). Characterization of an amorphous materials hybrid polymer electrolyte based on a LiNO_3_-doped, CMC-PVA blend for application in an electrical double layer capacitor. Mater. Chem. Phys..

[105] Aziz S.B., Abdullah O.G., Rasheed M.A. (2017). Structural and electrical characteristics of PVA:NaTf based solid polymer electrolytes: Role of lattice energy of salts on electrical DC conductivity. J. Mater. Sci. Mater. Electron..

[106] Arof A.K., Kufian M.Z., Syukur M.F., Aziz M.F., Abdelrahman A.E., Majid S.R. (2012). Electrical double layer capacitor using poly(methyl methacrylate)-C_4_BO_8_Li gel polymer electrolyte and carbonaceous material from shells of mata kucing (*Dimocarpus longan*) fruit. Electrochim. Acta.

[107] Sampathkumar L., Christopher Selvin P., Selvasekarapandian S., Perumal P., Chitra R., Muthukrishnan M. (2019). Synthesis and characterization of biopolymer electrolyte based on tamarind seed polysaccharide, lithium perchlorate and ethylene carbonate for electrochemical applications. Ionics (Kiel).

[108] Liew C.W., Ramesh S., Arof A.K. (2014). Good prospect of ionic liquid based-poly(vinyl alcohol) polymer electrolytes for supercapacitors with excellent electrical, electrochemical and thermal properties. Int. J. Hydrogen Energy.

[109] Shuhaimi N.E.A., Alias N.A., Majid S.R., Arof A.K. (2009). Electrical double layer capacitor with proton conducting Κ-Carrageenan–Chitosan electrolytes. Funct. Mater. Lett..

